# Tacrolimus Exposure is Associated with Acute Rejection in the Early Phase After Kidney Transplantation: A Joint Modeling Approach

**DOI:** 10.1097/FTD.0000000000001359

**Published:** 2025-07-25

**Authors:** Maaike R. Schagen, Alvaro Assis de Souza, Karin Boer, Jesse H. Krijthe, Rachida Bouamar, Andrew P. Stubbs, Dennis A. Hesselink, Brenda C.M. de Winter

**Affiliations:** * Division of Nephrology and Transplantation, Department of Internal Medicine, Erasmus MC Transplant Institute, University Medical Center Rotterdam, Rotterdam, the Netherlands;; † Rotterdam Clinical Pharmacometrics Group, Rotterdam, the Netherlands;; ‡ Pattern Recognition & Bioinformatics Group, Delft University of Technology, Delft, the Netherlands;; § Department of Hospital Pharmacy, Erasmus MC, University Medical Center Rotterdam, Rotterdam, the Netherlands; and; ¶ Department of Pathology and Clinical Bioinformatics, Erasmus MC Stubbs Group, University Medical Center Rotterdam, Rotterdam, the Netherlands.

**Keywords:** tacrolimus, biopsy-proven allograft rejection, joint models

## Abstract

**Background:**

Reports regarding the relationship between tacrolimus exposure and the risk of acute kidney allograft rejection are conflicting. This may be explained by the previous use of methodological approaches that disregarded important factors in the analysis of longitudinal measurements and time-to-event data. Therefore, in this study, joint models were used to investigate the relationship between repeated measurements of tacrolimus predose concentrations (C_0_) and time to acute biopsy-proven acute rejection (BPAR).

**Methods:**

This was a post hoc analysis of a randomized controlled trial in which living-donor kidney transplant recipients (KTR) received either a standard, bodyweight-based or *CYP3A5* genotype-based tacrolimus starting dose. Joint modeling was performed by coupling a mixed-effects model for tacrolimus C_0_ with a Cox proportional hazards model for the risk of rejection. Only the first episode of rejection was considered.

**Results:**

A total of 229 KTRs were included, of whom the incidence of BPAR was 10.5% (n = 24 KTRs) in the first 3 months posttransplant. A total of 3069 tacrolimus measurements were available for the analysis. A joint model adjusted for recipient age and peak panel reactive antibodies demonstrated that tacrolimus C_0_ was associated with risk of rejection. A 1-unit increase in the time-normalized area under the curve for logarithmically (log)-transformed C_0_ represented a change of −2.65 in the log of the relative hazard (95% credible interval: −5.05 to −0.36, *P* = 0.022).

**Conclusions:**

A negative association between the cumulative effect of tacrolimus C_0_ and BPAR was observed using joint modeling. This demonstrated that KTRs with lower tacrolimus exposure were at a higher risk of rejection.

## Abstract 2

Supplemental Digital Content is Available in the Text.

## INTRODUCTION

Therapeutic drug monitoring (TDM) is a standard practice for the immunosuppressant tacrolimus,^1^ in which whole blood predose concentrations (C_0_) are measured to personalize treatment by dose adjustments.^2^ This is particularly useful because tacrolimus and acute kidney allograft rejection seem to have an exposure–response relationship, in which lower exposure to tacrolimus is associated with a higher risk of acute rejection.^3–9^ Nonetheless, a lack of this exposure–response relationship has also been observed during the first 6 months after transplantation.^10^

These conflicting findings regarding the exposure–response relationship between tacrolimus C_0_ and allograft rejection could be explained by the use of methodological approaches that disregarded important factors in the analysis of longitudinal measurements and time-to-event data. First, longitudinal repeated measurements of tacrolimus C_0_ are often analyzed as a single mean or median of the observed values. However, tacrolimus is known for its highly variable pharmacokinetics over time, owing to factors, such as the cytochrome P450 (CYP) genotype,^11–16^ changes in hematocrit or serum albumin,^17^ or drug–drug interactions.^18,19^ The intrapatient variability (IPV), which denotes tacrolimus C_0_ fluctuations within an individual over time when the tacrolimus dose is left unchanged, is of particular importance.^20^ Patients with a high IPV are at risk for worse transplant outcomes,^21–25^ and patients with a higher time-in-therapeutic range have superior outcomes posttransplant.^26–28^ Thus, not considering the temporal evolution of tacrolimus C_0_ in the analysis could result in loss of information regarding the association between tacrolimus C_0_ and the risk of allograft rejection. Second, time-varying tacrolimus C_0_ is frequently included as a covariate in Cox proportional hazards models for time-to-event data. However, these models do not appropriately address the characteristics of time-varying covariates, such as tacrolimus C_0_, measurement errors induced by biological variation, and the fact that the C_0_ is only known at the time of measurement and not between measurement time points.^29,30^

Another common strategy in the biomedical field is to use separate models to analyze longitudinal (tacrolimus C_0_) and time-to-event (rejection) data. However, the dependence between both outcomes is then neglected, and this could lead to increased biases in the results.^31^ By using a joint modeling strategy, one can determine the association between repeated tacrolimus C_0_ measurements and rejection while considering the dependence among measurements from an individual patient.^30^ Meziyerh et al^32^ used a joint modeling approach to assist in determining long-term target ranges for tacrolimus and mycophenolic acid exposure in kidney transplant recipients (KTR). This study showed that immunosuppressant concentrations measured repeatedly between 1 and 3 years after transplantation are associated with the risk of developing biopsy-proven acute rejection (BPAR).^32^ Evidently, the exposure–response relationship between tacrolimus C_0_ and allograft rejection in the early period after kidney transplantation has never been analyzed using joint modeling. Therefore, this study primarily aimed to investigate this relationship by using an appropriate modeling technique for joint analysis of repeated tacrolimus C_0_ measurements and time to kidney allograft rejection. The secondary goal was to assess the relationship between tacrolimus C_0_ and posttransplant diabetes mellitus (PTDM).

## MATERIALS AND METHODS

### Study Design and Patients

This study was a post hoc analysis of data collected from KTRs in the first 3 months posttransplantation, who were included in a randomized-controlled, open-label, single-center clinical trial.^33^ This trial included patients ≥18 years, who were scheduled to receive a single-organ, blood group AB0-compatible kidney from a living donor at the Erasmus MC, University Medical Center, Rotterdam, the Netherlands. This trial aimed to assess whether a *CYP3A5* genotype-based starting dose was more favorable than a standard bodyweight-based starting dose in earlier achievement of the target tacrolimus whole blood C_0_. Between November 2010 (first patient, first visit) and September 2013 (last patient, first visit), patients were randomly assigned to receive tacrolimus (Prograf, Astellas Pharma, Leiden, the Netherlands). Patients either received a standard, bodyweight-based dose of 0.20 mg/kg/d^34^ or a dose determined by their *CYP3A5* genotype. Carriers of 1 or 2 CYP3A5*1 alleles (expressers) received 0.30 mg/kg/d, whereas CYP3A5*3 homozygotes (nonexpressers) received 0.15 mg/kg/d. This study was conducted in accordance with the Declaration of Helsinki and approved by the Medical Ethical Review Board of Erasmus MC (MEC number 2010-080). Written informed consent was obtained from all the participants before inclusion and randomization.

### Data Collection and Clinical Phenotypes

Demographic data and clinical characteristics were prospectively collected during the trial and used for post hoc analysis. Kidney allograft biopsies were only performed for cause, were blindly reviewed by 2 independent pathologists once the trial was completed, and were graded according to the Banff 2013 classification of that time.^35^ The study only included BPAR episodes.

PTDM was defined as the use of glucose-lowering medical therapy until month 3 posttransplantation by a recipient not needing such treatment before transplantation.^33^

### Tacrolimus Analysis

Tacrolimus was administered at 10:00 and 22:00 hours. Target tacrolimus concentrations were defined as: 10–15 ng/mL for the first 15 days, 8–12 ng/mL from days 15–28, and 5–10 ng/mL from day 28 onwards. According to the institutional protocol, the first tacrolimus C_0_ was measured on day 3 posttransplantation, which was considered the first steady state (5 times its half-life), after the tacrolimus doses were tailored using TDM. At the study center, tacrolimus C_0_ is measured 3 times per week during hospitalization and at every outpatient clinic visit (scheduled weekly in the first 2 months posttransplantation). All tacrolimus concentrations were measured at the ISO15189-certified central hospital laboratory using 2 methods. Samples from 2000 to 2012 were measured with an antibody-conjugated magnetic immunoassay, and those from 2013 were measured with an enzyme-multiplied immunoassay. Samples with values >30 or <1.5 ng/mL were either excluded (if the sample was not considered C_0_) or set to values of 30 and 1.5 ng/mL, respectively, if they were the true C_0_ values. Similarly, for samples without the exact time of measurement, it was assumed that it occurred just before morning dose ingestion (eg, 09:45 hours).

### Additional Immunosuppressive Treatment

All patients were administered 20 mg of basiliximab intravenously (i.v.) on days 0 and 4 for induction therapy. Patients also received mycophenolate mofetil at a starting dose of 1000 mg twice daily, aiming for plasma mycophenolic acid C_0_ values between 1.5 and 3.0 mg/L. Prednisolone (50 mg i.v. twice daily) was administered on days 0–3, followed by 20 mg once per day orally on days 4–14, and tapered to 5 mg at month 3 posttransplantation. Patients with BPAR were treated with pulse methylprednisolone (1000 mg i.v.) for 3 consecutive days and with T-lymphocyte-depleting antibody therapy in cases of severe or steroid-resistant rejection. Patients with antibody-mediated rejection were treated with pulsed methylprednisolone and intravenous immunoglobulin.

### Genotyping

Genotyping of the CYP3A5*3 allele was performed during the transplantation in an ISO15189-certified laboratory. DNA was extracted from the peripheral blood leukocytes using a Blood DNA kit (Qiagen, Courtaboeuf, France). For more information, please refer to the original trial.^33^

### Statistical Analysis

Continuous variables are reported as median (interquartile range). Categorical variables are presented as proportions and percentages.

Joint modeling was performed by coupling a mixed-effects model for tacrolimus C_0_ with a Cox proportional hazards model for the risk of kidney allograft rejection. The mixed-effects model (longitudinal sub-model) was used to estimate and reconstruct the full time-wise trajectory of tacrolimus C_0_ for each patient. This allowed for the estimation of its association with the risk of rejection using the Cox proportional hazard model (survival submodel).^30^ Only the first occurrence of allograft rejection was considered. Tacrolimus concentrations measured after the first BPAR episode were excluded from the analysis.

For the longitudinal submodel, logarithmically (log) transformed tacrolimus C_0_ was used as the dependent variable to ensure the normality of residuals and constant variance across fitted values. The independent variables (fixed effects) were *time* and *recipient age*. Natural cubic splines with 2 degrees of freedom were used to model the nonlinear relationship between *time* and tacrolimus C_0_. Random intercepts and either a linear term or 2 degrees-of-freedom splines for *time* were considered random effects. By assessing model improvement using Bayesian Information Criteria, the final longitudinal submodel included 2-degrees-of-freedom cubic splines for the random effect of *time*. This strategy allowed for different starting points and flexible evolution of tacrolimus C_0_ in each patient. No multiple imputation strategy was considered for the few samples with capped longitudinal outcome values because this strategy has shown no added value for linear mixed-effects models.^36^

The following 3 sets of independent variables were considered for the survival submodel: (1) *recipient age*; (2) *recipient age* and *peak panel reactive antibodies (PRA)*; and (3) *recipient age*, *peak PRA*, and *total number of human leukocyte antigen (HLA) mismatches*. Through log-likelihood ratio tests, variable set (2) was chosen to be included in the model. These tests were performed before the joint modeling. Both submodels were corrected for age, with previously reported effects on both longitudinal and survival outcomes. Given the modeling limitations imposed by the relatively small number of rejection events, no further variables were included in this model. The proportional hazards assumption was verified by assessing the relationship between Schoenfeld residuals and time.

The joint model was then fitted and the association between the full-time course of tacrolimus C_0_ and the risk of rejection was estimated. The cumulative effect of log-transformed tacrolimus C_0_ was used as the association structure linking both submodels, that is, the normalized area under the curve of log-transformed tacrolimus C_0_. This transformation was selected because the risk of rejection was most likely driven by proportional changes rather than absolute differences in tacrolimus C_0_.

As a secondary outcome, the association between tacrolimus exposure and the occurrence of PTDM was examined. The same cumulative association structure using the log-transformed tacrolimus C_0_ values was applied. As in the rejection model, the longitudinal submodel included age and 2-degree-of-freedom spline terms for *time* as fixed effects and random intercepts and 2-degree-of-freedom spline terms for *time* as random effects. In the survival submodel for PTDM occurrence, *age* and *lean bodyweight* were included as covariates.

In addition to joint modeling analysis, the association between tacrolimus C_0_ and BPAR was assessed using 3 other previously reported methods.^5,10^ First, the Mann–Whitney *U* test was used to compare tacrolimus C_0_ values between the BPAR and no-BPAR group at the following 4 different time points: day 3 (±2 days), day 10 (±2 days), day 14 (±3 days), and 1 month (±7 days) posttransplantation.^10^ The other 2 approaches were as follows: (1) application of binary logistic regression fitted to the patient-level median tacrolimus C_0_ and (2) addition of the time-varying tacrolimus C_0_ as a covariate in a Cox proportional hazards model.^5,10^ Both models were corrected for *recipient age* and *peak PRA*, as done in the survival submodel for joint modeling.

All statistical analyses were performed using R statistical software (version 4.4.1), RStudio (version 2024.04.2 + 764), and the R packages *JMbayes2* (version 0.5-0), *dplyr* (version 1.1.4), and *ggplot2* (version 3.5.1). Statistical significance was defined as a Bayesian *P*-value <0.05. Additional methodological information is available in the Supplementary Materials (see **Text**, **Supplemental Digital Content 1**, http://links.lww.com/TDM/A864, which further describes the statistical analysis).

## RESULTS

### Study Population

A total of 237 living-donor KTRs were included, of whom 230 patients completed the 3 months of follow-up.^33^ For this analysis, 1 patient was excluded because measurements were only available after having experienced allograft rejection. Thus, 229 KTRs were included in the analysis, of whom 116 (51%) received a bodyweight-based starting dose and 113 (49%) received a genotype-based starting dose. In the first 3 months after transplantation, the incidence of BPAR was 10.5% (n = 24 KTRs). The baseline patient characteristics are shown in Table 1.

**TABLE 1.** Patient Characteristics

Patient Characteristics	Kidney Transplant Recipients (n = 229)
Recipient	
Sex (male/female)	141 (62%)/88 (38%)
Age (years)	56 (46–64)
Bodyweight (kg)	78.8 (66.7–91.6)
Length (cm)	173 (165–181)
BMI (kg/m^2^)	25.9 (23.4–29.5)
Primary kidney disease (n)	
Diabetic nephropathy	44 (19%)
Polycystic kidney disease	34 (15%)
Glomerulonephritis	43 (19%)
Hypertensive nephropathy	28 (12%)
Reflux disease/chronic pyelonephritis	12 (5%)
Other	8 (4%)
Unknown	60 (26%)
Transplantation	
RRT prior to transplantation (n, %)	
None	99 (43)
Peritoneal dialysis	42 (18)
Hemodialysis	88 (39)
Donor type (living-related/living-unrelated)	93 (41%)/136 (59%)
Number of kidney transplantations (n, %)	
1	211 (92)
2	16 (7)
3	2 (1)
HLA mismatch (n)	3 (3–5)
Current PRA (%)	0 (0–4)
Peak PRA (%)	4 (0–5)
Cold ischemic period (h)	2 (2–2)
Donor	
Sex (male/female)	112 (49%)/117 (51%)
Age (yrs)	53 (42–62)
Tacrolimus exposure	
Dosing	
Bodyweight-based dosing (n, %)	116 (51)
Genotype-based dosing (n, %)	
*CYP3A5* expressers	31 (14)
*CYP3A5* nonexpressers	82 (36)
Tacrolimus starting dose (mg/kg)	
Bodyweight-based	7.5 (6.5–9.0)
*CYP3A5* expressers	12.0 (11.0–13.0)
*CYP3A5* nonexpressers	6.0 (5.5–7.0)

Demographic and clinical characteristics of 229 kidney transplant recipients and donors at transplantation time. Data are shown as the number of patients (percentage) for categorical variables and median (interquartile range) for continuous variables.

BMI, body mass index; HLA, human leukocyte antigen; RRT, renal replacement therapy.

### Trajectory of Tacrolimus Exposure

In total, 3069 tacrolimus C_0_ measurements were available for analysis. Eight samples had values >30 ng/mL, of which 5 were excluded because they were not considered true predose concentrations (patients were already taking the medication), and 3 were set at 30 ng/mL. One sample was <1.5 ng/mL and was considered a true low concentration as the dose was increased; this sample was set to 1.5 ng/mL and included in the analysis. The median number of measurements per patient during follow-up was 13 (11–16). The median follow-up time was 89.8 (84.9–92.9) days after transplantation. The median first tacrolimus C_0_ was 11.5 (8.1–17.0) ng/mL and was measured at 90.3 (66.4–91.2) hours after transplantation.

**FIGURE 1.**
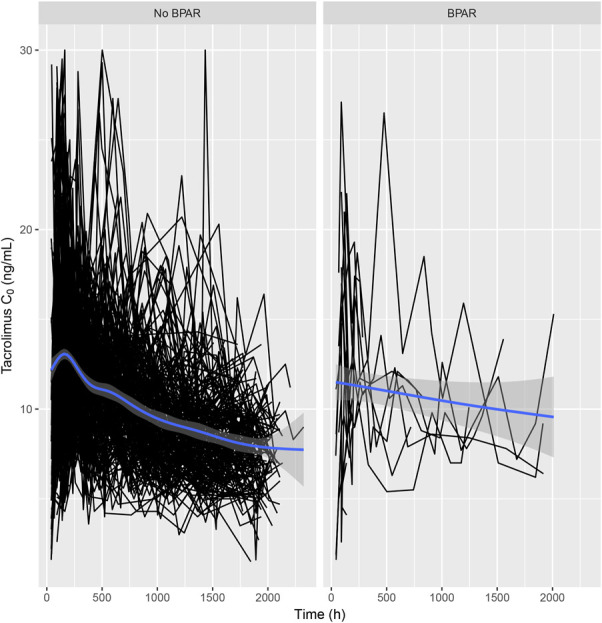
Time-wise trajectory of tacrolimus C_0_ divided into recipients with and without biopsy-proven acute rejection (A) and depicting the values of all recipients (B). BPAR, biopsy-proven acute rejection; C0, predose concentration.

When comparing KTRs with and without BPAR, both groups showed an overall decrease in tacrolimus C_0_ over time (Fig. 1). In the BPAR group, the median number of tacrolimus measurements per patient leading up to the rejection episode was 2 (2.0–8.3). For these patients, the median follow-up time was 7.8 (5.8–37.3) days after transplantation, and the median first value of measured tacrolimus C_0_ was 11.0 (6.9–17.2) ng/mL and was measured 90.3 (58.9–90.9) hours after transplantation.

### Joint Modeling of Allograft Rejection

A joint model, adjusted for *recipient age* and *peak PRA*, demonstrated that repeated measurements of tacrolimus C_0_ are associated with the onset of acute allograft rejection. A 1-unit increase in the time-normalized area under the curve for the log-transformed C_0_ represented a change of −2.65 in the log of the relative hazard (95% credible interval: −5.05 to −0.36, *P* = 0.022). Simply, a 1-unit decrease in the time-normalized area under the curve for log-transformed tacrolimus C_0_ (ie, the geometric mean of tacrolimus C_0_ was 37% of the actual value) increased the risk of acute allograft rejection 14-fold. A summary of the model results is presented in Table 2.

**TABLE 2.** Joint Model for Tacrolimus and Kidney Allograft Rejection

Parameter	Mean	SD	95% Credible Interval	*P*
Survival outcome				
Age	0.0299	0.0230	–0.0126 to 0.0767	0.1864
Peak PRA	0.0202	0.0072	0.0054 to 0.0335	0.0092
Area (log(tacrolimus C_0_))	−2.6516	1.1841	–5.0525 to −0.3606	**0.0224**
Longitudinal outcome (log(tacrolimus C_0_) (family = Gaussian, link = identity)				
Intercept	2.4356	0.0420	2.3534 to 2.5180	<0.0001
First spline term for time after transplantation	−0.6211	0.0545	–0.7277 to −0.5133	<0.0001
Second spline term for time after transplantation	−0.4824	0.0371	–0.5555 to −0.4095	<0.0001
Age	0.0012	0.0007	–0.0001 to 0.0025	0.0719
Sigma	0.3035	0.0043	0.2952 to 0.3120	<0.0001

*P*-value for the variable of interest is shown in bold.

C_0_, predose concentration; PRA, panel reactive antibodies.

### Joint Modeling of Posttransplant Diabetes Mellitus

**FIGURE 2.**
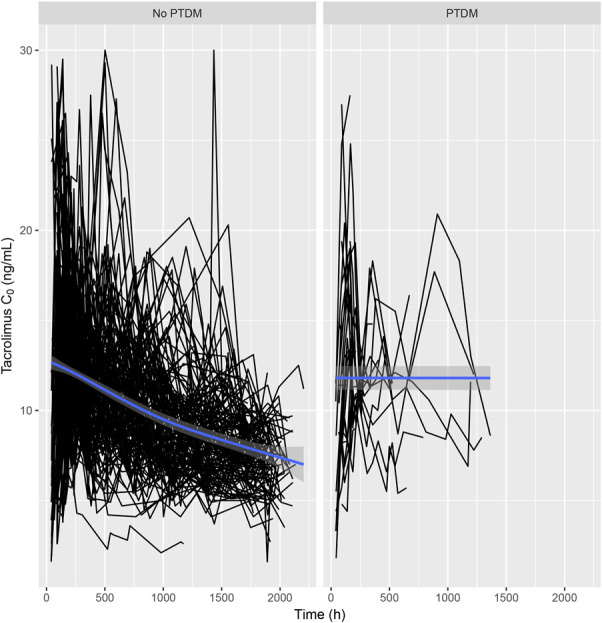
Time-wise trajectory of tacrolimus C_0_ split for patients with and without PTDM (A) and depicting the values of all recipients (B). C0, predose concentration.

Subanalysis identified an exposure–response relationship between tacrolimus and PTDM. A total of 44 patients with preexisting diabetes mellitus were excluded, and another 7 were excluded because their first measured tacrolimus C_0_ was after the onset of PTDM. Therefore, 178 KTRs were available for this analysis. When comparing KTRs with and without PTDM, both groups showed an overall decrease in tacrolimus C_0_ over time (Fig. 2). The joint model, adjusted for *recipient age* and *lean bodyweight*, did not demonstrate an association between repeated tacrolimus C_0_ and PTDM occurrence. A 1-unit increase in the time-normalized area under the log-transformed tacrolimus C_0_ curve represented a change of −0.26 in the log of the relative hazard (95% credible interval: −2.4 to 2.1, *P* = 0.8). Simply, a 1-unit decrease in the time-normalized area under the curve for log-transformed tacrolimus C_0_ (ie, the geometric mean of tacrolimus C_0_ was 37% of the actual value) increased the risk of PTDM occurrence by 30% (nonsignificant). A summary of the model results is presented in Table 3.

**TABLE 3.** Joint Model for Tacrolimus and Posttransplant Diabetes Mellitus

Parameter	Mean	SD	95% Credible Interval	*P*
Survival outcome				
Age	0.0241	0.0177	–0.0096 to 0.0586	0.1741
Lean bodyweight	−0.0091	0.0228	–0.0543 to 0.0349	0.6907
Area(log(tacrolimus C_0_))	−0.2628	1.1340	–2.3583 to 2.0601	**0.7923**
Longitudinal outcome (log(tacrolimus C_0_)) (family = Gaussian, link = identity)				
Intercept	2.3979	0.0499	2.3007 to 2.4963	0.0000
First spline term for time after transplantation	−0.5382	0.0652	–0.6676 to −0.4120	0.0000
Second spline term for time after transplantation	−0.5175	0.0352	–0.5861 to −0.4483	0.0000
Age	0.0013	0.0008	–0.0003 to 0.0030	0.1152
Sigma	0.2995	0.0049	0.2901 to 0.3094	0.0000

*P*-value for the variable of interest is shown in bold.

C_0_, predose concentration.

### Additional Analysis for BPAR

No significant association between tacrolimus exposure and the risk of BPAR was observed in any additional analysis performed. These analyses included the Mann–Whitney U tests at 4 time points, a logistic regression model, and a Cox proportional hazards model with time-varying tacrolimus C_0_ as a covariate. All analyses are presented in the Supplementary Materials (see **Fig. S4 and Text**, **Supplemental Digital Content 1**, http://links.lww.com/TDM/A864, which further present the results of these analyses).

## DISCUSSION

In this post hoc analysis of 229 KTRs, a joint modeling approach revealed that KTRs with lower tacrolimus exposure were at a higher risk for biopsy-proven kidney allograft rejection in the first 3 months after transplantation. More specifically, a cumulative effect of tacrolimus C_0_ on the risk of allograft rejection in the first 3 months posttransplantation was demonstrated. The joint model could discriminate KTRs who experienced BPAR from those who did not in the first 90 days after transplantation using tacrolimus measurements from the first 3 days after transplantation [Supplementary Materials (see **Fig. S3**, **Supplemental Digital Content 1**, http://links.lww.com/TDM/A864, which further presents the result of this analysis)].

The identified association between tacrolimus concentrations and onset of rejection is in line with previous research.^3–9^ For example, Israni et al*^5^* investigated 1930 KTRs and revealed that for each 1 ng/mL decrease in tacrolimus C_0_, a 7.2% increase in the risk of acute rejection in the first 6 months posttransplant was observed [hazards ratio (HR) = 1.07; 95% confidence interval: 1.01–1.14; *P* = 0.03]. This was analyzed by use of a Cox proportional hazard model including time-varying tacrolimus C_0_ as a covariate. In addition, Gaynor et al^6^ analyzed 528 KTRs and found that for each 1 ng/mL increase in tacrolimus C_0_, a 18.0% lower BPAR rate was observed in the first 12 months posttransplant (HR –0.199; standard error ±0.056; *P* = 0.0003). Moreover, they recommended maintaining the tacrolimus C_0_ at >4.0 ng/mL.^6^ Similarly, the study used a Cox proportional hazard model, in which the most recent tacrolimus C_0_ was used prior or equal to a certain time point (7 and 14 days, and 1, 2, 3, 6, and 9 months) posttransplantation for each KTR.^6^

By contrast, Bouamar et al*^10^* investigated 1304 KTRs who participated in 3 randomized controlled trials (RCT)^37–39^ and found that the tacrolimus C_0_ was not significantly associated with the risk of developing BPAR in the first 6 months posttransplantation (odds ratio: 0.98; 95% confidence interval: 0.94 to 1.03; *P* = 0.48). In this analysis, a binary logistic regression was performed in which the tacrolimus C_0_ for each KTR was converted into a single median value. Although these 3 aforementioned studies investigated larger patient cohorts than this study, the statistical methods used fell short when considering repeated measurements from the same patients and the use of the full temporal evolution of tacrolimus values. As additional evidence of the potential information loss with these methods, the supplementary analyses in this study conducted using similar approaches found no significant association between tacrolimus exposure and the risk of BPAR. However, the joint modeling approach is especially appropriate when repeated tacrolimus measurements are performed at variable timepoints and the number of available repeated measurements is unequal between KTRs,^30^ which is usually the case when using real-world data. As tacrolimus C_0_ may change at a certain moment in time, the joint model approach results in less bias than that of simplified models.

The study findings suggest that it is important to reach the therapeutic tacrolimus concentration as soon as possible after transplantation and maintain the concentration within the therapeutic range as much as possible. The tacrolimus starting dose is often based on the patient's body weight.^40^ However, by using this approach, only 18.5%–37.4% of KTRs will have a first tacrolimus C_0_ measurement within the therapeutic range.^33,41,42^ In this post hoc analysis, only 60 KTRs (26%) were within the target range of 11–15 ng/mL at the first C_0_ measurement. For the KTRs who experienced BPAR, only 5 (21%) were within the target range of 11–15 ng/mL at the first C_0_ measurement. Although the median first tacrolimus C_0_ measurement in these KTRs was 11.0 ng/mL (the lowest boundary of the target range), the KTRs still experienced BPAR, and a significant association between tacrolimus exposure and the risk of BPAR was identified. The confounders of recipient age and peak PRA, which are known to affect the risk of BPAR, were corrected for. However, other variables, such as exposure to additional immunosuppressants, cytomegalovirus status, sex, and HLA mismatches, also contribute to this risk.^43^ Therefore, starting dose algorithms should consider numerous variables instead of body weight alone, thereby limiting the time of tacrolimus exposure outside the therapeutic range.^44,45^

In the subanalysis of the relationship between tacrolimus and the occurrence of diabetes, 32 KTRs (22%) developed PTDM; however, no significant association was observed between tacrolimus exposure and the occurrence of PTDM. However, the incidence of PTDM could vary because of concomitant risk factors and several other factors that were not accounted for.^46^

This study has some limitations. First, a relatively small sample size was used, with a limited number of rejection events. This constrains the inclusion of additional covariates in the model. In addition, the sample size could explain the large effect size of the area under the log-transformed tacrolimus C_0_ curve on the risk of rejection. Second, this was a post hoc analysis of an RCT performed between 2010 and 2013. Since then, several advances, including the updated Banff classification for allograft rejection, have been made in the medical field. Hence, the results of this RCT could possibly differ in light of the new classifications.^47^ Furthermore, many centers have now switched to liquid chromatography-tandem mass spectrometry-based methods for the analysis of tacrolimus, which is more specific than the antibody-conjugated magnetic immunoassay and enzyme-multiplied immunoassay that were used in this analysis.^48^ However, this post hoc analysis is relevant for clinical care and furthermore emphasizes the use of appropriate statistical methods. Third, only tacrolimus C_0_ was included, whereas a measured area under the concentration versus time curve or peak concentrations could provide more information on tacrolimus exposure.^49^ Furthermore, the target range for tacrolimus has changed since the trial was performed, with most centers now targeting slightly lower C_0_. However, this aspect will probably not influence the results in this study.

## CONCLUSION

By using an approach that more effectively uses both longitudinal tacrolimus data and the occurrence of rejection, this study provided further evidence for the negative association between tacrolimus exposure and the risk of acute kidney allograft rejection. Accordingly, future studies should use joint modeling techniques to clarify these associations.

## Supplementary Material
