# Supplementary material for: Tacrolimus Exposure is Associated with Acute Rejection in the Early Phase After Kidney Transplantation: A Joint Modeling Approach
Source: Ther Drug Monit. 2025 Jul 25;47(6):e82–9. doi: 10.1097/FTD.0000000000001359 (PMC12588658; doi:10.1097/FTD.0000000000001359)
Supplement: Supplementary file 1 [file tdm-47-e82-s001.docx]

**Supplementary Appendix Material**

·

TACROLIMUS EXPOSURE IS ASSOCIATED WITH ACUTE REJECTION IN THE EARLY PHASE AFTER KIDNEY TRANSPLANTATION: A JOINT MODELING APPROACH

·

Maaike R. Schagen, MD^1,2,#^, Alvaro Assis de Souza, MSc^1,#^, Karin Boer, PhD^1^, Jesse H. Krijthe, PhD^3^, Rachida Bouamar, PharmD, PhD^4^, Andrew P Stubbs, PhD^5^, Dennis A. Hesselink, MD, PhD^1^, Brenda C.M. de Winter, PharmD, PhD^2,4^

Table of Contents

[**Methods** 3](#_Toc193310514)

[**Results – Tacrolimus and rejection** 4](#_Toc193310515)

[**Figure S1.** Standardized residuals against the fitted values - longitudinal model 4](#_Toc193310516)

[**Figure S2.** Q-Q plot of residuals - longitudinal model 4](#_Toc193310517)

[**Table S1.** Schoenfeld residuals test for proportionality of hazards – survival model 5](#_Toc193310518)

[**Figure S3.** Time varying ROC-Curve 5](#_Toc193310519)

[**Summary of joint model** 6](#_Toc193310520)

[**Summary of survival sub-model** 6](#_Toc193310521)

[**Summary of longitudinal sub-model** 7](#_Toc193310522)

[**Anova table (log-likelihood ratio test) for selection of survival model** 7](#_Toc193310523)

[**Figure S4.** Mann-Whitney U test 8](#_Toc193310524)

[**Summary of logistic regression** 9](#_Toc193310525)

[**Summary of Cox proportional hazards model** 9](#_Toc193310526)

[**Results – Tacrolimus and post-transplant diabetes mellitus** 10](#_Toc193310527)

[**Figure S4.** Standardized residuals against the fitted values - longitudinal model 10](#_Toc193310528)

[**Figure S5.** Q-Q plot of residuals - longitudinal model 10](#_Toc193310529)

[**Table S2.** Schoenfeld residuals test for proportionality of hazards – survival model 11](#_Toc193310530)

[**Figure S6.** Time varying ROC-Curve 11](#_Toc193310531)

[**Summary of joint model** 12](#_Toc193310532)

[**Summary of survival sub-model** 12](#_Toc193310533)

[**Summary of longitudinal sub-model** 13](#_Toc193310534)

# **Methods**

Model discrimination analysis: To certify that both joint models learned meaningful signal from the data, their apparent discrimination ability was checked via a time-dependent receiver operator characteristic curve (ROC) analysis. The area under the ROC curve was assessed by using tacrolimus measurements from the first three days after transplantation to predict the occurrence of both endpoints in the next three months.

# **Results – Tacrolimus and rejection**

## **Figure S1.** Standardized residuals against the fitted values - longitudinal model

**
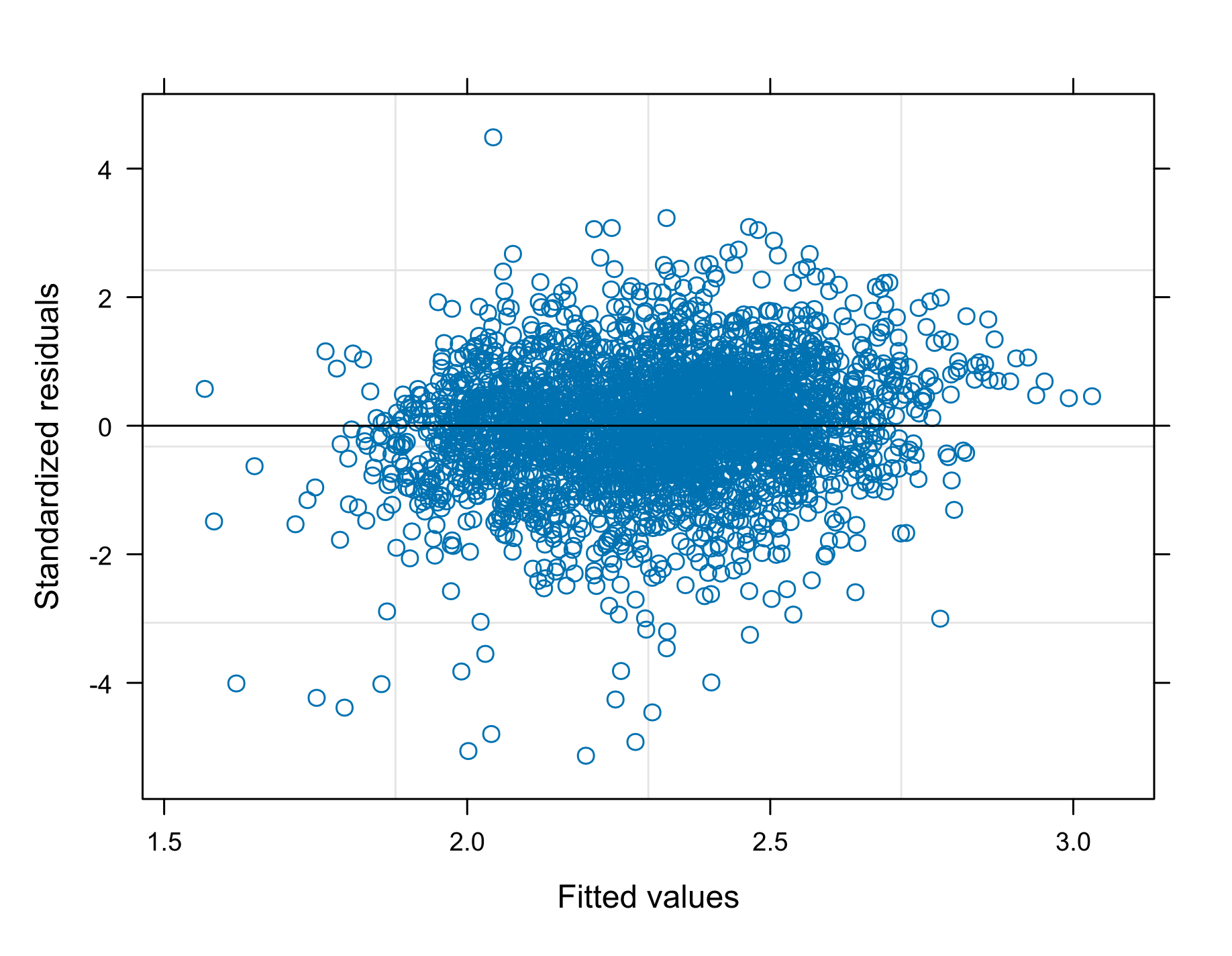
**

## **Figure S2.** Q-Q plot of residuals - longitudinal model

**
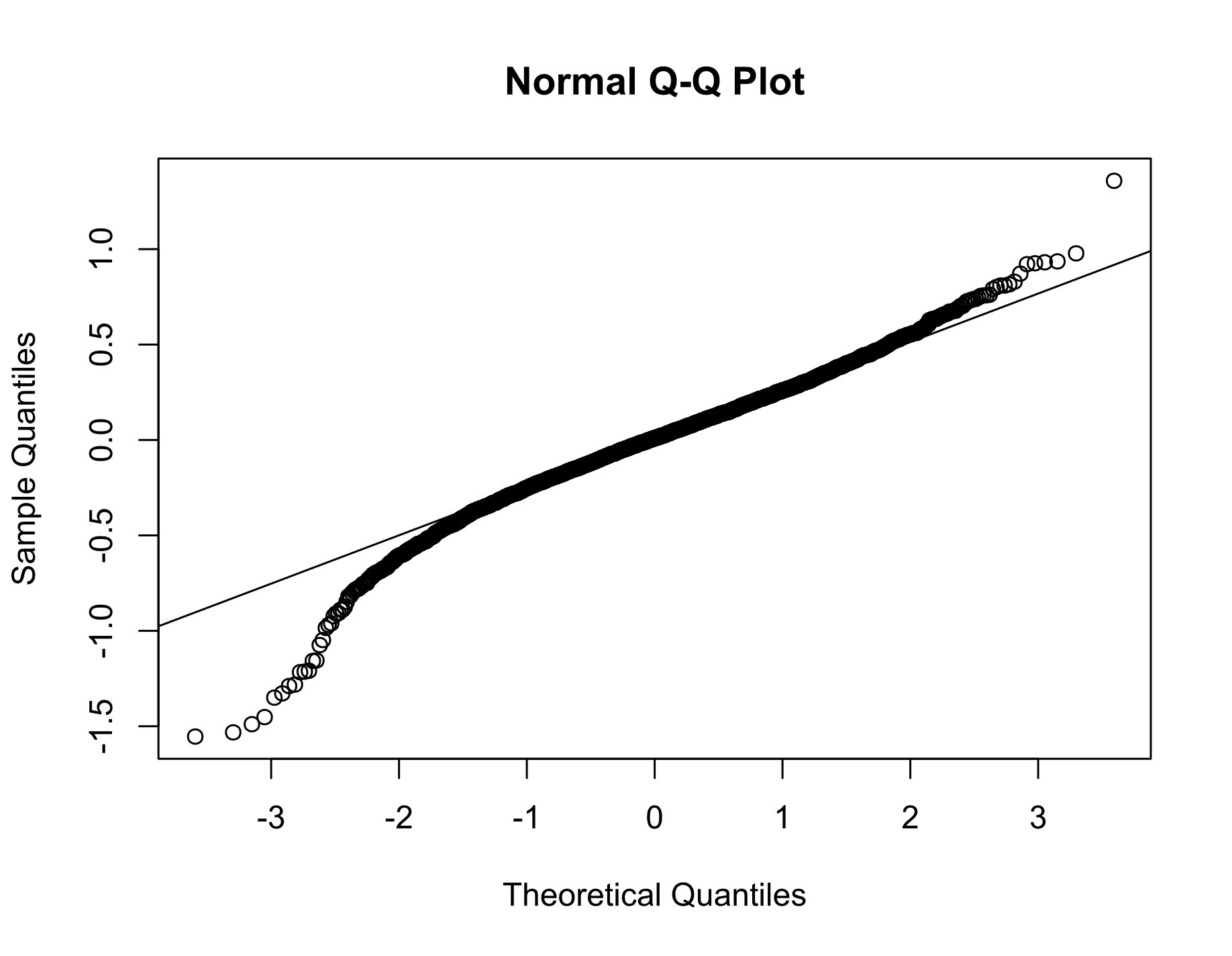
**

## **Table S1.** Schoenfeld residuals test for proportionality of hazards – survival model

|  | **Chi-square** | **df** | **p-value** |
| --- | --- | --- | --- |
| **AGE** | **0.7853** | **1** | **0.38** |
| **High_PRA** | **0.0364** | **1** | **0.85** |
| **GLOBAL** | **0.7853** | **2** | **0.68** |

## **Figure S3.** Time varying ROC-Curve

**
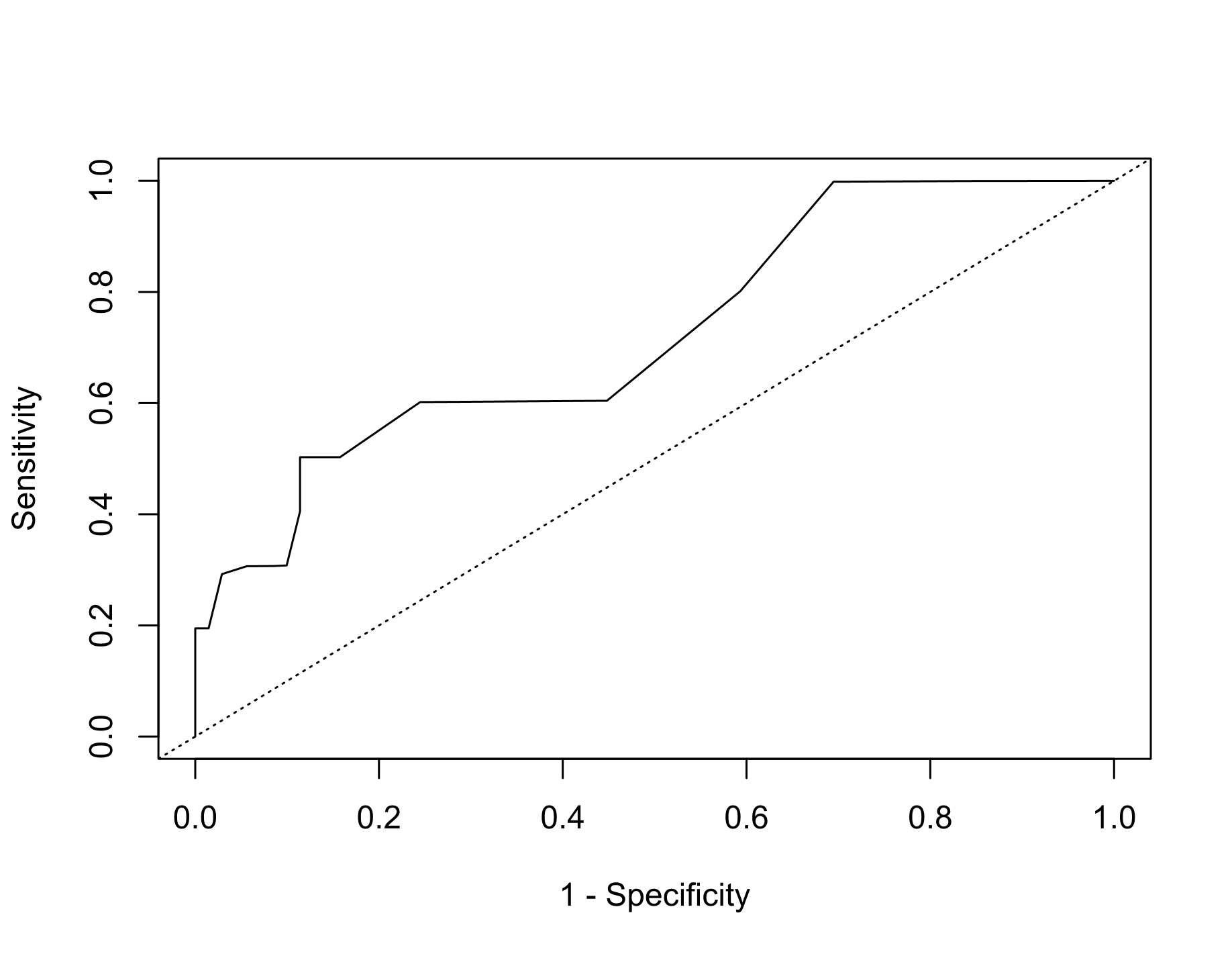
**

AUC-ROC: 0.72

At time: 2232

For the 79 subjects at risk at time 72

Number of subjects with an event in [72, 2232): 10

Number of subjects with a censored time in [72, 2232): 53

Accounting for censoring using model-based weights

## **Summary of joint model**

Data Descriptives:

Number of Groups: 229 Number of events: 24 (10.5%)

Number of Observations:

log(DV.TCL): 3069

DIC WAIC LPML

marginal 2392.852 2417.712 -1212.635

conditional 2579.043 2420.223 -1488.168

Random-effects covariance matrix:

StdDev Corr

(Intr) 0.2608 (Intr) n(TIM2

n(TIME_,2)1 0.6689 -0.9068

n(TIME_,2)2 0.2433 0.1678 -0.2272

Survival Outcome:

Mean StDev 2.5% 97.5% P Rhat

AGE 0.0299 0.0230 -0.0126 0.0767 0.1864 1.0023

High_PRA 0.0202 0.0072 0.0054 0.0335 0.0092 1.0040

area(log(DV.TCL)) -2.6516 1.1841 -5.0525 -0.3606 0.0224 1.0010

Longitudinal Outcome: log(DV.TCL) (family = gaussian, link = identity)

Mean StDev 2.5% 97.5% P Rhat

(Intercept) 2.4356 0.0420 2.3534 2.5180 0.0000 1.0007

ns(TIME_meas, 2)1 -0.6211 0.0545 -0.7277 -0.5133 0.0000 1.0013

ns(TIME_meas, 2)2 -0.4824 0.0371 -0.5555 -0.4095 0.0000 1.0003

AGE 0.0012 0.0007 -0.0001 0.0025 0.0719 1.0002

sigma 0.3035 0.0043 0.2952 0.3120 0.0000 1.0046

MCMC summary:

chains: 4

iterations per chain: 25000

burn-in per chain: 10000

thinning: 1

time: 2.7 min

## **Summary of survival sub-model**

Call:

coxph(formula = Surv(TIME, status2) ~ AGE + High_PRA, data = df_wide)

n= 229, number of events= 24

coef exp(coef) se(coef) z Pr(>|z|)

AGE 0.022632 1.022890 0.015992 1.415 0.157

High_PRA 0.025363 1.025688 0.006429 3.945 7.97e-05 ***

---

Signif. codes: 0 ‘***’ 0.001 ‘**’ 0.01 ‘*’ 0.05 ‘.’ 0.1 ‘ ’ 1

exp(coef) exp(-coef) lower .95 upper .95

AGE 1.023 0.9776 0.9913 1.055

High_PRA 1.026 0.9750 1.0128 1.039

Concordance= 0.663 (se = 0.056 )

Likelihood ratio test= 13.04 on 2 df, p=0.001

Wald test = 15.84 on 2 df, p=4e-04

Score (logrank) test = 18.53 on 2 df, p=9e-05

## **Summary of longitudinal sub-model**

Linear mixed-effects model fit by REML

Data: df

AIC BIC logLik

1956.771 2023.076 -967.3853

Random effects:

Formula: ~ns(TIME_meas, 2) | STNR

Structure: General positive-definite, Log-Cholesky parametrization

StdDev Corr

(Intercept) 0.2672955 (Intr) n(TIM2

ns(TIME_meas, 2)1 0.6892913 -0.921

ns(TIME_meas, 2)2 0.2562255 0.229 -0.293

Residual 0.3027033

Fixed effects: log(DV.TCL) ~ ns(TIME_meas, 2) + AGE

Value Std.Error DF t-value p-value

(Intercept) 2.4413223 0.03985193 2838 61.25983 0.0000

ns(TIME_meas, 2)1 -0.6303183 0.05542318 2838 -11.37283 0.0000

ns(TIME_meas, 2)2 -0.4807574 0.03700137 2838 -12.99296 0.0000

AGE 0.0011812 0.00062573 227 1.88769 0.0603

Correlation:

(Intr) n(TIME_,2)1 n(TIME_,2)2

ns(TIME_meas, 2)1 -0.486

ns(TIME_meas, 2)2 0.086 -0.168

AGE -0.845 0.005 0.021

Standardized Within-Group Residuals:

Min Q1 Med Q3 Max

-5.13360515 -0.54011466 0.03125022 0.58892610 4.48733227

Number of Observations: 3069

Number of Groups: 229

## **Anova table (log-likelihood ratio test) for selection of survival model**

Analysis of Deviance Table

Cox model: response is Surv(TIME, status2)

Model 1: ~ AGE

Model 2: ~ AGE + High_PRA

Model 3: ~ AGE + High_PRA + mm.total

loglik Chisq Df Pr(>|Chi|)

1 -127.64

2 -121.54 12.2039 1 0.0004769 ***

3 -121.28 0.5269 1 0.4679133

---

Signif. codes: 0 ‘***’ 0.001 ‘**’ 0.01 ‘*’ 0.05 ‘.’ 0.1 ‘ ’ 1

## **Figure S4.** Mann-Whitney U test


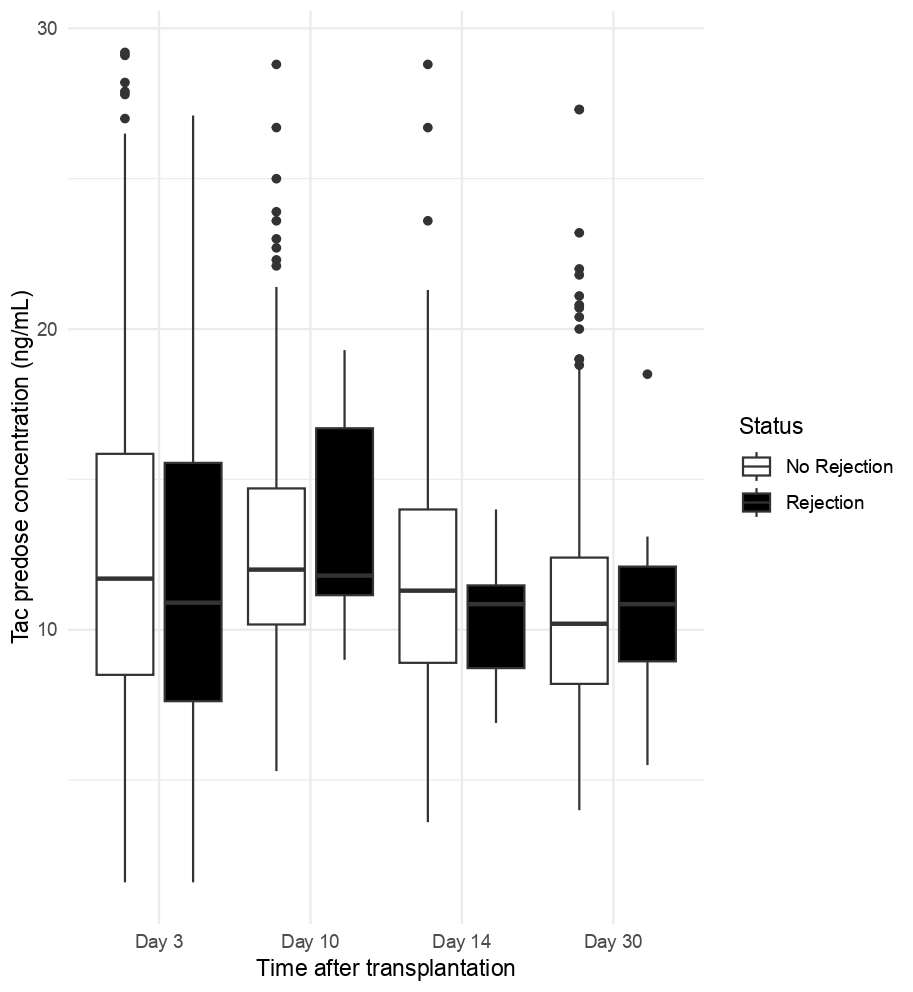


As can be appreciated from the figure above, no significant association between tacrolimus exposure and the risk of BPAR was observed for all time-points (Mann-Whitney U Test): Day 3: W = 5251, p-value = 0.29; Day 10: W = 2289.5, p-value = 0.29; Day 14: W = 2361, p-value = 0.21; Day 30: W = 4065, p-value = 0.70.

## **Summary of logistic regression**

glm(formula = status2 ~ median_DV_TCL + AGE + High_PRA, family = binomial,

data = df_wide)

Coefficients:

Estimate Std. Error z value Pr(>|z|)

(Intercept) -5.406134 1.617333 -3.343 0.000830 ***

median_DV_TCL 0.139390 0.115317 1.209 0.226755

AGE 0.023248 0.018209 1.277 0.201694

High_PRA 0.030651 0.008177 3.748 0.000178 ***

---

Signif. codes: 0 ‘***’ 0.001 ‘**’ 0.01 ‘*’ 0.05 ‘.’ 0.1 ‘ ’ 1

(Dispersion parameter for binomial family taken to be 1)

Null deviance: 153.66 on 228 degrees of freedom

Residual deviance: 139.50 on 225 degrees of freedom

AIC: 147.5

Number of Fisher Scoring iterations: 5

## **Summary of Cox proportional hazards model**

coxph(formula = Surv(tstart, tstop, endpt) ~ DV.TCL + Age + High_PRA,

data = tm1)

n= 3068, number of events= 24

coef exp(coef) se(coef) z Pr(>|z|)

DV.TCL -0.070730 0.931714 0.050922 -1.389 0.1648

Age 0.025806 1.026142 0.016297 1.583 0.1133

High_PRA 0.023851 1.024138 0.006413 3.719 0.0002 ***

---

Signif. codes: 0 ‘***’ 0.001 ‘**’ 0.01 ‘*’ 0.05 ‘.’ 0.1 ‘ ’ 1

exp(coef) exp(-coef) lower .95 upper .95

DV.TCL 0.9317 1.0733 0.8432 1.030

Age 1.0261 0.9745 0.9939 1.059

High_PRA 1.0241 0.9764 1.0113 1.037

Concordance= 0.677 (se = 0.05 )

Likelihood ratio test= 14.92 on 3 df, p=0.002

Wald test = 17.69 on 3 df, p=5e-04

Score (logrank) test = 20.29 on 3 df, p=1e-04

# **Results – Tacrolimus and post-transplant diabetes mellitus**

## **Figure S4.** Standardized residuals against the fitted values - longitudinal model

**
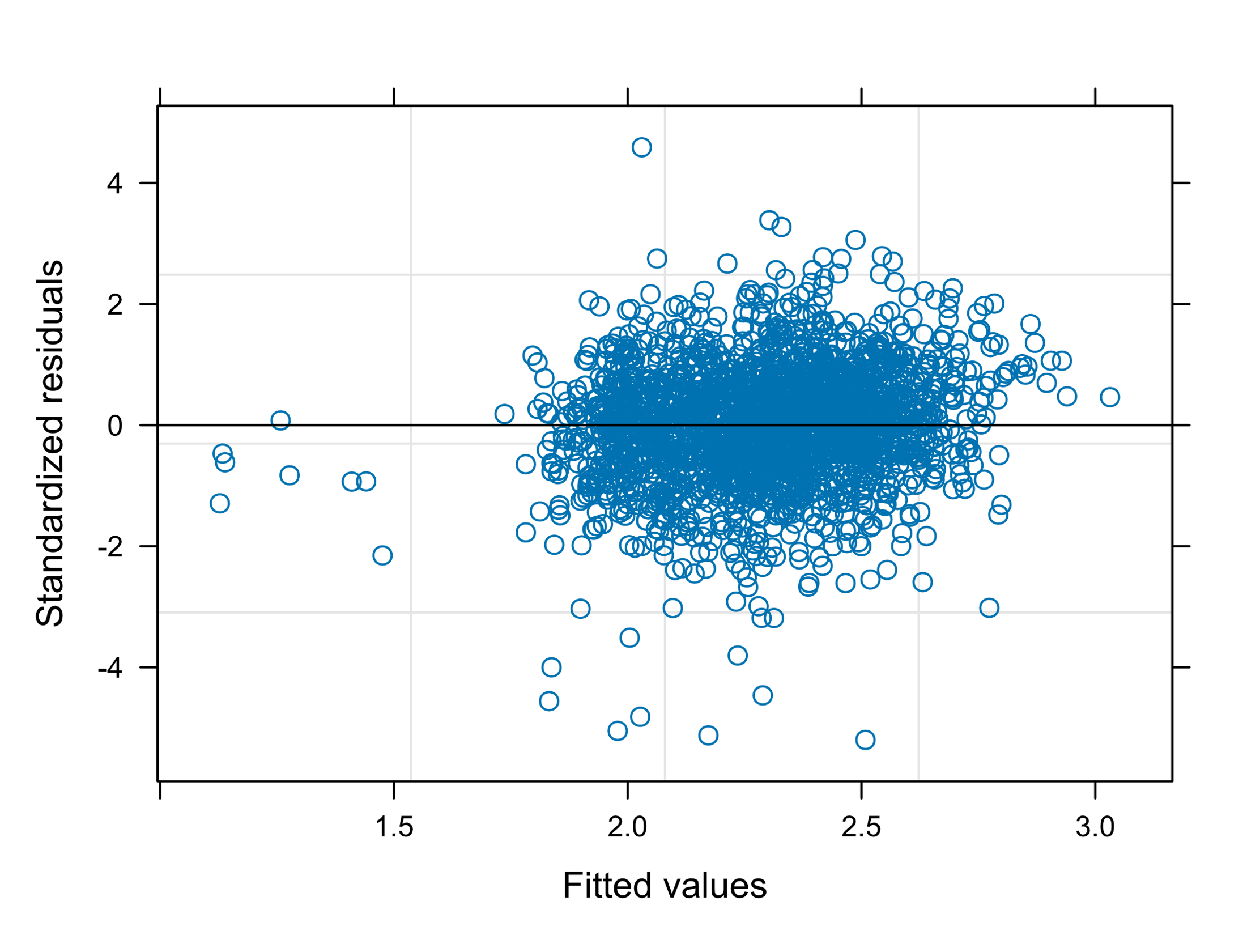
**

## **Figure S5.** Q-Q plot of residuals - longitudinal model

**
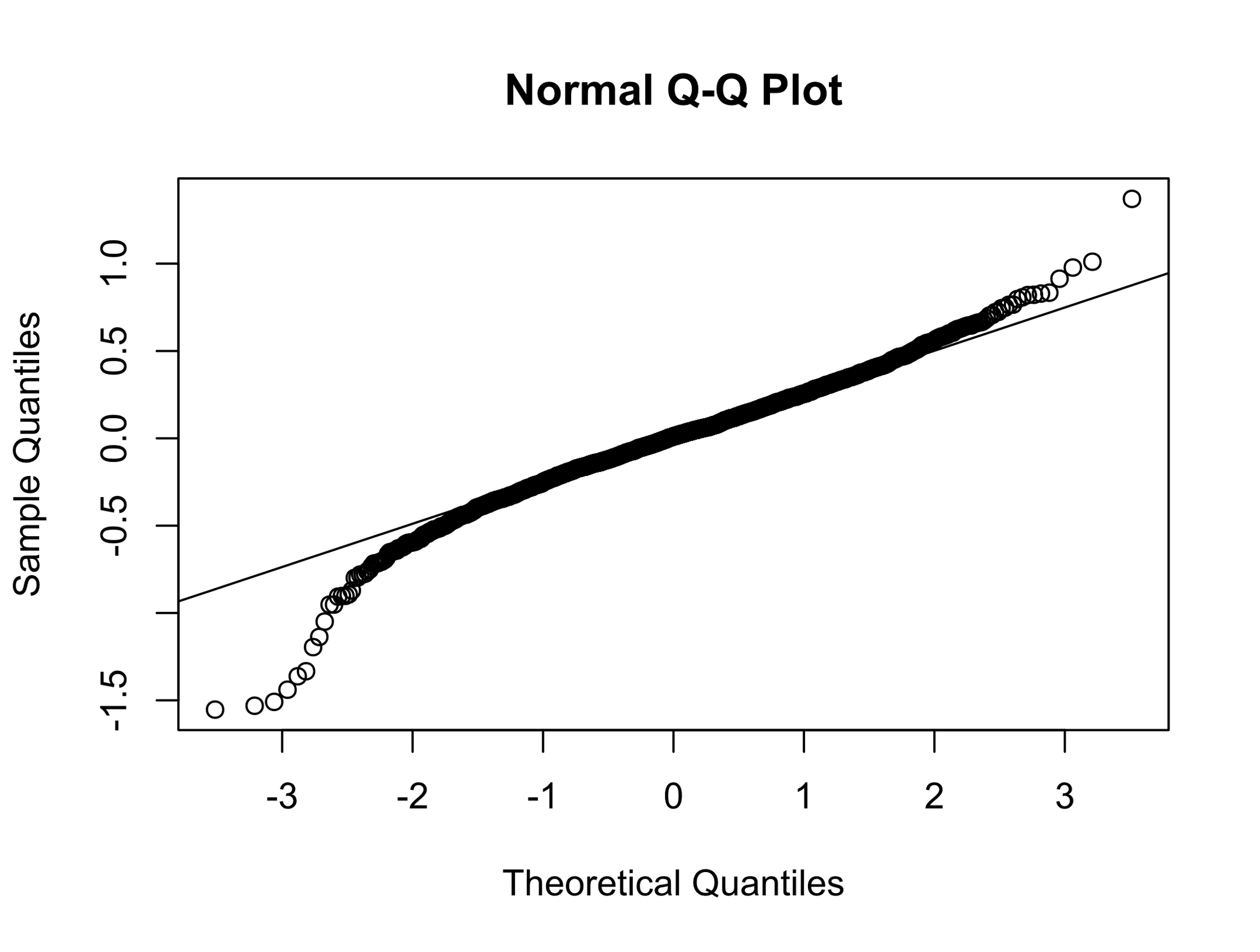
**

## **Table S2.** Schoenfeld residuals test for proportionality of hazards – survival model

|  | **Chi-square** | **df** | **p-value** |
| --- | --- | --- | --- |
| **AGE** | **0.28044** | **1** | **0.60** |
| **LBW** | **0.00623** | **1** | **0.94** |
| **GLOBAL** | **0.28071** | **2** | **0.87** |

## **Figure S6.** Time varying ROC-Curve

**
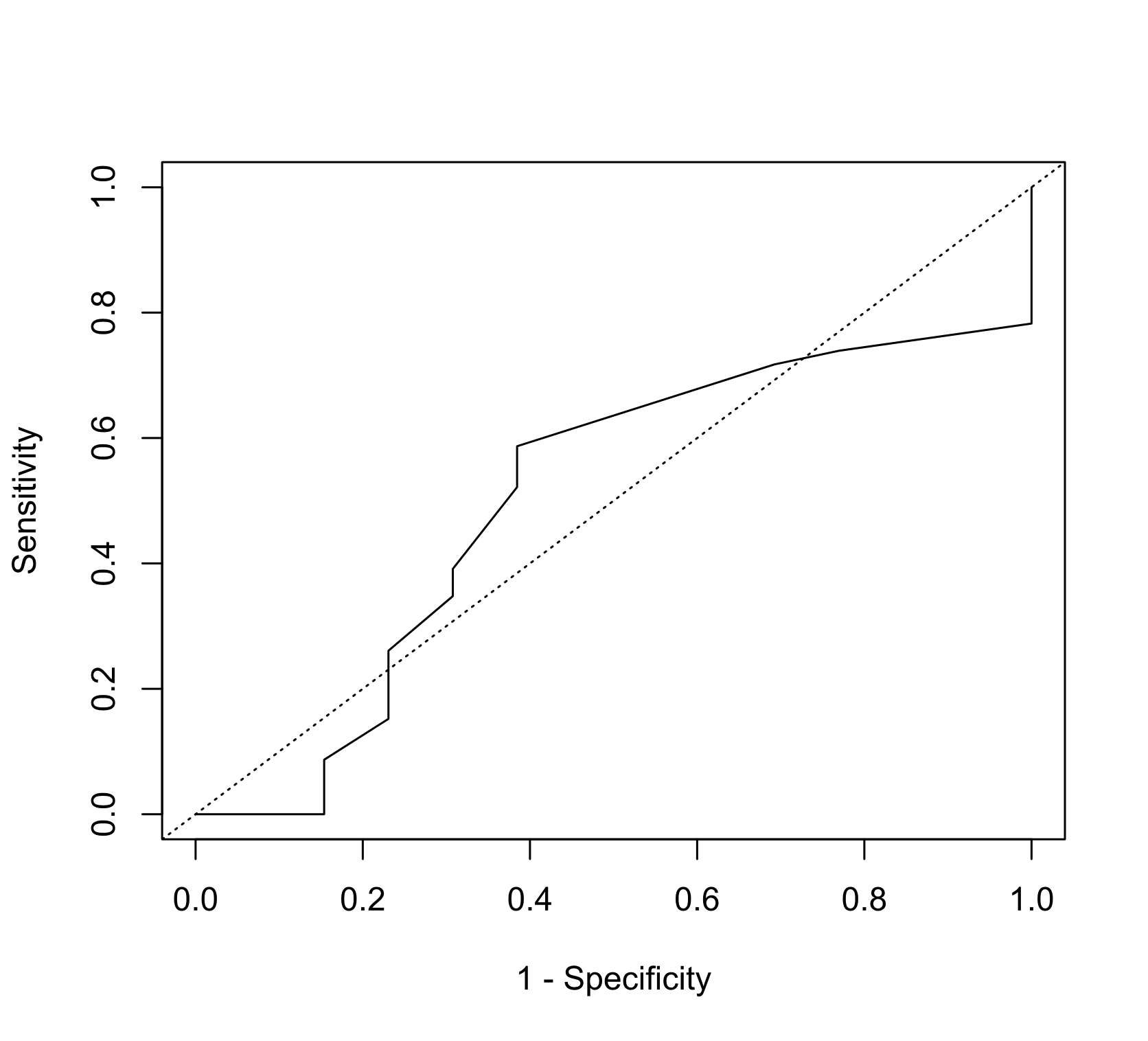
**

Estimated AUC: 0.58

At time: 2232

Using information up to time: 72 (59 subjects still at risk)

Accounting for censoring using model-based weights

## **Summary of joint model**

Data Descriptives:

Number of Groups: 178 Number of events: 32 (18%)

Number of Observations:

log(DV.TCL): 2269

DIC WAIC LPML

marginal 2014.793 2115.075 -1121.597

conditional 2029.431 1951.131 -1207.453

Random-effects covariance matrix:

StdDev Corr

(Intr) 0.2648 (Intr) n(TIM2

n(TIME_,2)1 0.7033 -0.8597

n(TIME_,2)2 0.1807 0.0570 -0.3072

Survival Outcome:

Mean StDev 2.5% 97.5% P Rhat

AGE 0.0241 0.0177 -0.0096 0.0586 0.1741 1.0012

LBW -0.0091 0.0228 -0.0543 0.0349 0.6907 1.0031

area(log(DV.TCL)) -0.2628 1.1340 -2.3583 2.0601 0.7923 1.0061

Longitudinal Outcome: log(DV.TCL) (family = gaussian, link = identity)

Mean StDev 2.5% 97.5% P Rhat

(Intercept) 2.3979 0.0499 2.3007 2.4963 0.0000 1.0018

ns(TIME_meas, 2)1 -0.5382 0.0652 -0.6676 -0.4120 0.0000 1.0011

ns(TIME_meas, 2)2 -0.5175 0.0352 -0.5861 -0.4483 0.0000 1.0014

AGE 0.0013 0.0008 -0.0003 0.0030 0.1152 1.0011

sigma 0.2995 0.0049 0.2901 0.3094 0.0000 1.0008

MCMC summary:

chains: 4

iterations per chain: 25000

burn-in per chain: 10000

thinning: 1

time: 2.2 min

## **Summary of survival sub-model**

coxph(formula = Surv(tx_hours_to_onset, status2) ~ AGE + LBW,

data = df_wide)

n= 178, number of events= 32

coef exp(coef) se(coef) z Pr(>|z|)

AGE 0.02264 1.02290 0.01299 1.744 0.0812 .

LBW -0.01020 0.98985 0.02100 -0.486 0.6272

---

Signif. codes: 0 ‘***’ 0.001 ‘**’ 0.01 ‘*’ 0.05 ‘.’ 0.1 ‘ ’ 1

exp(coef) exp(-coef) lower .95 upper .95

AGE 1.0229 0.9776 0.9972 1.049

LBW 0.9899 1.0103 0.9499 1.031

Concordance= 0.593 (se = 0.046 )

Likelihood ratio test= 3.23 on 2 df, p=0.2

Wald test = 3.07 on 2 df, p=0.2

Score (logrank) test = 3.11 on 2 df, p=0.2

## **Summary of longitudinal sub-model**

Linear mixed-effects model fit by REML

Data: df

AIC BIC logLik

1444.704 1507.683 -711.3522

Random effects:

Formula: ~ns(TIME_meas, 2) | STNR

Structure: General positive-definite, Log-Cholesky parametrization

StdDev Corr

(Intercept) 0.2742528 (Intr) n(TIM2

ns(TIME_meas, 2)1 0.7303072 -0.887

ns(TIME_meas, 2)2 0.2036986 0.108 -0.415

Residual 0.2987697

Fixed effects: log(DV.TCL) ~ ns(TIME_meas, 2) + AGE

Value Std.Error DF t-value p-value

(Intercept) 2.4029709 0.04712033 2089 50.99648 0.0000

ns(TIME_meas, 2)1 -0.5383347 0.06685361 2089 -8.05244 0.0000

ns(TIME_meas, 2)2 -0.5167256 0.03622473 2089 -14.26444 0.0000

AGE 0.0012354 0.00077464 176 1.59478 0.1126

Correlation:

(Intr) n(TIME_,2)1 n(TIME_,2)2

ns(TIME_meas, 2)1 -0.472

ns(TIME_meas, 2)2 0.041 -0.217

AGE -0.853 0.018 0.028

Standardized Within-Group Residuals:

Min Q1 Med Q3 Max

-5.19830135 -0.53771955 0.03729593 0.58015288 4.58861953

Number of Observations: 2269

Number of Groups: 178
